# Supplementary material for: Cannabinoid CB2 receptor ligand profiling reveals biased signalling and off-target activity
Source: Nat Commun. 2017 Jan 3;8:13958. doi: 10.1038/ncomms13958 (PMC5216056; doi:10.1038/ncomms13958)
Supplement: Supplementary Information — Supplementary Figures, Supplementary Tables, Supplementary Methods, Supplementary References. [file ncomms13958-s1.pdf]

Supplementary Figures

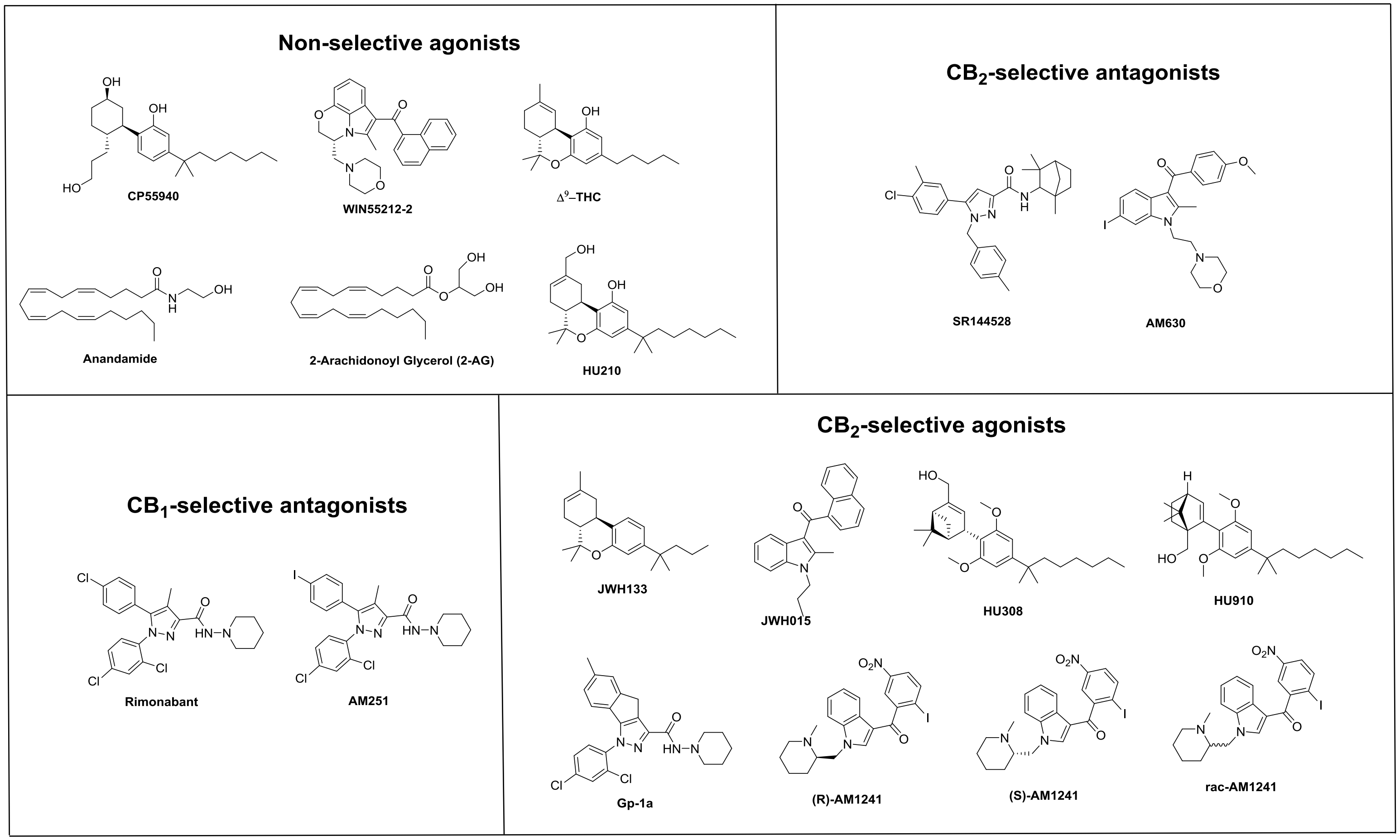

Supplementary Figure 1. Structures of reference ligands divided per class as described in the introduction

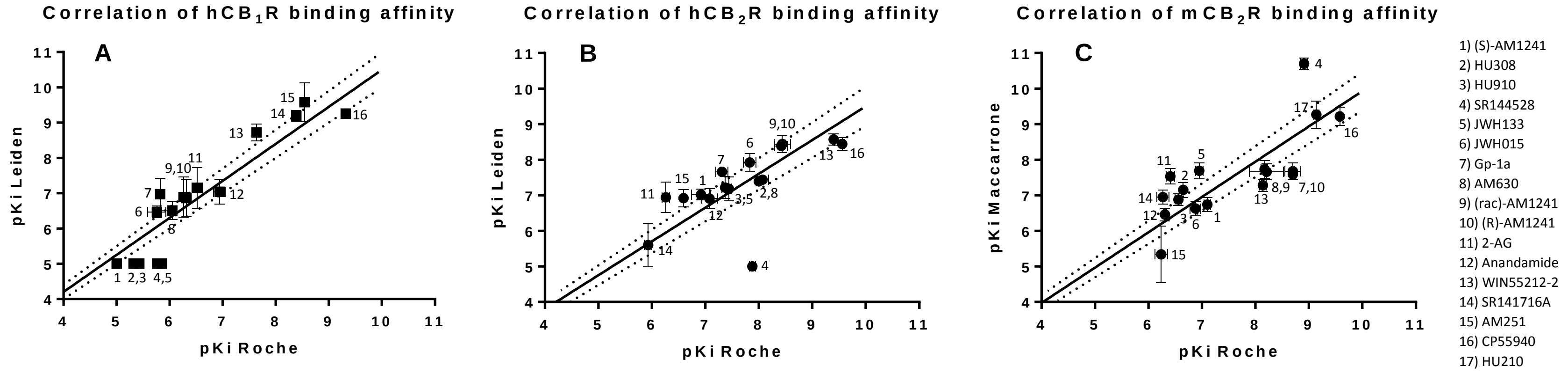

Supplementary Figure 2. Correlation of binding affinities between labs. The numbers on the dots indicates which reference ligand it belongs to, which can be found in the list of ligands shown on the right. It should be noted that the affinities of THC and HU210 could not be compared since these were not measured in all labs due to legal restrictions. Two numbers separated by a comma indicate two overlapping dots. A-B) Binding affinities of reference compounds separately determined by the labs of Roche and Leiden correlate well for hCB_1R (P-value <0.0001, Pearson coefficient: 0.9304) and hCB_2R (P-values < 0.005, Pearson coefficient: 0.6648). The only outlier is SR144528, which showed very low displacement on hCB_2R in the lab of Leiden for unknown reasons. C) Good correlation was found between mCB_2R binding affinity values separately obtained by the labs from Roche and Mauro Maccarrone (P-value 0.0003, Pearson coefficient: 0.7720). Statistics performed was two-tailed Pearson correlation analysis. All pKi values presented here are the mean ± SEM of three independent experiments performed in duplicate.

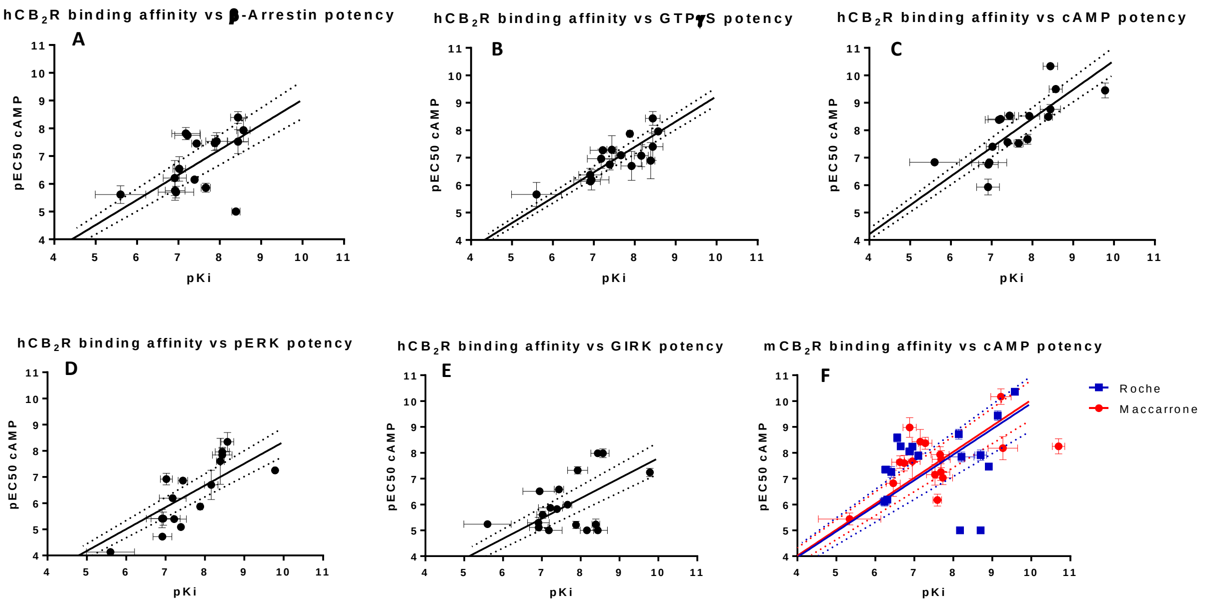

Supplementary Figure 3. Correlation between binding affinity and functional potency. (A-E) On hCB_2, binding affinities of the reference compounds in general correlate well with their functional potencies in all different assays, except in β-Arrestin and GIRK signaling, which may be because of biased signaling (P-values: β-arrestin 0.0074, GTPγS 0.0002, cAMP 0.0004, pERK 0.0002, GIRK 0.0536. Pearson coefficient: β-arrestin 0.4589, GTPγS 0.7931, cAMP 0.7629, pERK 0.7939, GIRK 0.4620). F) On mCB_2, there seems to be good correlation between all compounds in Maccarrone's data (P-value 0.0285, Pearson coefficient; 0.5305). There is a good correlation in Roche's data set, except for (Rac)-AM1241 and (R)-AM1241, which seem to be inactive in their mouse cAMP assay. When these two ligands are excluded from analysis, the P-value of correlation is 0.0069 (Pearson coefficient: 0.6647). Statistics performed was two-tailed Pearson correlation analysis. All pEC_{50} values presented here are the mean ± SEM of three independent experiments performed in duplicate.

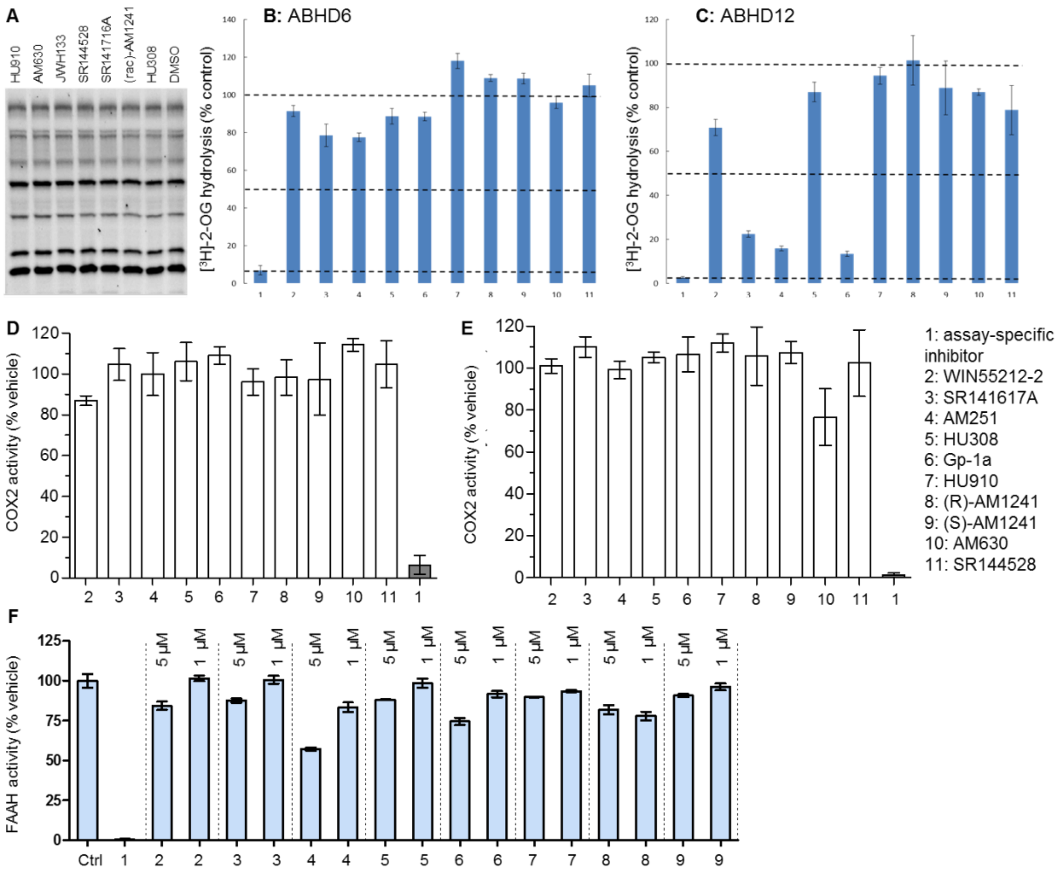

Supplementary Figure 4. Off-target activity of reference ligands on ECS proteins. A) None of the reference ligands showed any off-target activity on serine hydrolases up to a concentration of 10 µM in a competitive ABBP assay in mouse brain proteome. Shown is an example gel, with MB064 as the probe used in a concentration of 250 nM, as described previously in Baggelaar *et al*.[1] Gel experiments were repeated twice with independently weighed compounds stocks. B-C) Reference ligands were incubated with lysates of ABHD6- or ABHD12 overexpressing HEK293T cells. WWL70 (10 µM) and THL (20 µM) were used as the positive control **1** for ABHD6 and ABHD12, respectively. The values obtained for each measurement were corrected for non-specific hydrolysis (non-ABHD6 or ABHD12 mediated), which was calculated by substracting the basal [^{3}H]- 2-OG hydrolysis measured in non-transfected HEK293 cells. None of the compounds showed a significant inhibition of ABHD6 or ABHD12, except for SR141716A, Gp-1a and AM251. These compounds were tested in a full dose-response assay, of which the data is shown in Table 12. D-E) Specific inhibition of COX2 activity, using as a substrate either 10 µM AEA (D) or 10 µM 2-AG (E), was measured with

DuP-697 as the positive control **1**. None of the compounds showed a significant inhibition of the enzyme activity. F) FAAH inhibition was measured in U937 homogenate at 1 or 5 µM. None of the compounds showed a large effect at either concentration, except for AM251 and Gp-1a, which showed partial inhibition (~30-40%) at 5 µM. URB597 (1 µM) was used as the positive control **1**. Data shown in panels B-F are the mean ± SEM of two independent experiments performed in duplicate.

Supplementary Tables

Supplementary Table 1. Physicochemical properties of ligands.

	MW (g/mol)	Polar surface area[a] (Å)	Kow clogP[b]	logD[c, j]	Pampa Peff[d, j] [cm/s*10^{-6}]	Pct acceptor[e, j] [%]	Pct donor[f, j] [%]	Pct membrane[g, j] [%]	Kinetic solubility[h, k] [µg/mL]	Kinetic solubility[h, k] [µmol/mL]	Solubility in 15% DMSO, 85% PEG400[i, l] [µg/mL]	Solubility in 15% DMSO, 85% PEG400[i, l] [µmol/mL]
**WIN55212-2**	522.619	36.28	4.685	3.67±0.03	0.25±0.22	0.5	50.6	49	<0.3	<0.0006	1.68	0.0032
**CP55940**	376.585	49.35	7.498	ND	0	0	53.2	46.7	1.4±0	0.037	ND	ND
**Δ^9-THC**	386.57	38.06	7.986	ND	ND	ND	ND	ND	ND	ND	ND	ND
**HU-210**	386.567	38.06	7.986	precipitation	0.22	0	23	76	<0.2	<0.0005	ND	ND
**SR141716A**	463.794	42.1	6.085	>3.335	0.27±0.11	0.3	25.2	74.3	<0.733333	<0.0016	1.23	0.0027
**AM251**	555.249	42.15	6.608	ND	0	0	10.3	89.3	<0.3	<0.0005	1.57	0.0028
**JWH015**	327.425	15.51	6.462	ND	ND	ND	ND	ND	<0.2	<0.0006	1.2	0.0037
**JWH133**	312.494	7.91	8.458	>3 precipitation	1.86	2.3	36.1	61.7	<0.4	<0.0013	1.88	0.006
**HU308**	414.626	27.44	8.965	4.29	2.53	2.8	23.1	74.3	<0.75	<0.0018	2.06	0.005
**Gp-1a**	441.36	43.1	5.931	ND	0	0	29.7	70.3	<0.2	<0.0005	2.43	0.0055
**HU910**	414.621	31.35	9.001	out of range	0.45	1	42	57	<0.5	0.0012	ND	ND
**(rac)-AM1241**	503.334	62.47	5.726	3.66±0.03	1.86±0.62	3.3.	41.3	55.7	25±2	0.0497±0.004	ND	ND
**(R)-AM1241**	503.334	58.26	5.726	3.66	1.22	2.1	38.3	59.7	12	0.0238	ND	ND
**(S)-AM1241**	503.334	62.65	5.726	3.64	1.41	2.2	34	64	6	0.0119	2.37	0.0047
**AM630**	504.362	39.77	4.862	3.82	0.2	0.37	35.4	64.3	<0.3	<0.0006	1.54	0.0031
**SR144528**	476.061	36.11	9.154	>3	0	0	47	54	<0.2	<0.0004	1.64	0.0034
**Anandamide**	347.535	41.91	6.31	out of range	0.26	1.2	96.3	2.7	<1.1	<0.0032	ND	ND
**2-AG**	378.549	51.32	6.738	ND	ND	ND	ND	ND	<0.5	<0.0013	ND	ND

[a]Surface sum of all polar atoms in the molecule; [b]Calculated partition coefficient values (cLogP) from experimentally determined octanol-water partition coefficient values (Kow); [c]Distribution coefficient values; [d]Parallel artificial membrane permeability assay (PAMPA) was used to determine membrane permeation coefficient values (Peff); [e]Percentage of molecule that is able to act as a hydrogen bond acceptor; [f]Percentage of molecule that is able to act as a hydrogen bond donor; [g]Percentage of compounds found in membranes; [h]Solubility of the compound when diluted into aqueous environment from DMSO superstock; [i]Solubility of the compound in the formulation used for *in vivo* administration of the ligands; [j]Mean of three independent experiments; [k]Mean of two independent experiments; [l]Single experiment

Supplementary Table 2. Binding affinity and selectivity of reference ligands on human and mouse cannabinoid receptors. Results shown (pKi ± SEM) on hCB_1R and hCB_2R are from Leiden, except HU210 and SR144528, which are from Roche. Mouse data is from the lab of Mauro Maccarrone. Results shown are obtained from three independent experiments performed in duplicate, unless stated otherwise.

	hCB_2R	hCB_1R	CB_2R Selectivity*	mCB_2R	mCB_1R	CB_2R Selectivity*
WIN55212-2	8.57 ± 0.16	8.72 ± 0.24	1	7.28 ± 0.17	7.81 ± 0.15	0.3
CP55940	8.44 ± 0.18	9.26 ± 0.12	0.2	9.22 ± 0.26	8.8 ± 0.03	3
Δ^9-THC	8.16 ± 0.17	8.48 ± 0.08	0.5	ND	ND	
HU210	9.78 ± 0.04	9.55 ± 0.06	2	9.27 ± 0.38	8.96 ± 0.27	2
SR141716A	5.60 ± 0.61	9.19 ± 0.10	0.0003	6.95 ± 0.2	8.35 ± 0.06	0.04
AM251	6.92 ± 0.24	9.58 ± 0.21	0.002	5.34 ± 0.8	8.62 ± 0.07	0.001
JWH015	7.92 ± 0.26	6.47 ± 0.09	28	6.63 ± 0.21	5.94 ± 0.15	5
JWH133	7.18 ± 0.34	<5	153	7.69 ± 0.23	6.09 ± 0.13	40
HU308	7.44 ± 0.12	<5	278	7.15 ± 0.21	6.08 ± 0.14	12
Gp-1a	7.66 ± 0.11	6.97 ± 0.35	5	7.68 ± 0.23	6.37 ± 0.08	20
HU910	7.22 ± 0.31	<5	166	6.88 ± 0.17	6.14 ± 0.13	6
(rac)-AM1241	8.39 ± 0.10	6.89 ± 0.31	32	7.73 ± 0.25	5.91 ± 0.16	66
(R)-AM1241	8.44 ± 0.25	6.86 ± 0.46	38	7.59 ± 0.11	6.36 ± 0.13	17
(S)-AM1241	7.02 ± 0.15	<5	105	6.74 ± 0.2	6.48 ± 0.08	2
AM630	7.39 ± 0.02	6.51 ± 0.25	8	7.66 ± 0.23	5.6 ± 0.298	115
SR144528	7.88 ± 0.06	5.77 ± 0.09	129	10.7 ± 0.16	6.92 ± 0.2	6026
Anandamide	6.91 ± 0.28	7.04 ± 0.28#	1	6.46 ± 0.18	7.13 ± 0.06	0.2
2-AG	6.94 ± 0.43	7.15 ± 0.47#	1	7.53 ± 0.22	6.99 ± 0.29	3

*CB_2R selectivity was calculated as follows: 10^(pKi CB_2R-pKi CB_1R)

[#]Mean ± SEM of 4 independent experiments performed in duplicate

Supplementary Table 3. Functional potency, efficacy and selectivity in the GTPγS assay. Results shown are obtained from three independent experiments performed in duplicate.

	hCB_2R		hCB_1R		hCB_2R Selectivity*	mCB_2R	
	pEC_{50} ± SEM	Emax ± SEM	pEC_{50} ± SEM	Emax ± SEM		pEC_{50} ± SEM	Emax ± SEM
WIN55212-2	7.96 ± 0.04	49 ± 7	7.61 ± 0.10	49 ± 6	2	ND	ND
CP55940	8.43 ± 0.25	95 ± 4	8.33 ± 0.19	92 ± 5	1	7.79 ± 0.12	102 ± 1
Δ9-THC	7.07 ± 0.40	21 ± 3	8.88 ± 0.42	26 ± 6	0.02	ND	ND
HU210	ND	ND	ND	ND		ND	ND
SR141716A	5.66 ± 0.44	-28 ± 9	8.98 ± 0.20	-29 ± 7	0.0005	ND	ND
AM251	6.14 ± 0.11	-29 ± 2	9.07 ± 0.45	-25 ± 5	0.001	ND	ND
JWH015	6.70 ± 0.53	67 ± 16	<5	19 ± 2	51	ND	ND
JWH133	6.96 ± 0.35	61 ± 15	7.37 ± 0.16	20 ± 2	0.4	7.03 ± 0.14	103 ± 7
HU308	7.29 ± 0.51	97 ± 12#	<5	17 ± 9	193	6.83 ± 0.01	96 ± 3
Gp-1a	7.09 ± 0.07	-14 ± 5	<5	-7 ± 4	123	ND	ND
HU910	7.27 ± 0.12	61 ± 13	<5	-16 ± 4	185	7.27 ± 0.08	78 ± 4
(rac)-AM1241	6.89 ± 0.66	56 ± 9	8.48 ± 0. 09	42 ± 6	0.03	ND	ND
(R)-AM1241	7.40 ± 0.50	33 ± 2	6.78 ± 0.40	47 ± 8	4	ND	ND
(S)-AM1241	-	47 ± 5#	<5	9 ± 4		ND	ND
AM630	6.75 ± 0.20	-22 ± 2	<5	-1 ± 1	56	ND	ND
SR144528	7.87 ± 0.08	-29 ± 2	<5	10 ± 4	739	ND	ND
Anandamide	6.37 ± 0.24	31 ± 4#	6.35 ± 0.30	40 ± 8#	1	ND	ND
2-AG	6.19 ± 0.37	57 ± 11#	6.85 ± 0.56	71 ± 20#	0.2	ND	ND

*CB_2R selectivity was calculated as follows: 10^(pEC50 CB_2R-pEC50 CB_1R)

The effect of agonists is normalized to the effect of 10 µM CP55940

Negative Emax values represent inverse agonism

#Emax, but no plateau observed

Effect at 10 µM

Supplementary Table 4. Functional potency, efficacy and selectivity in the cAMP assay.

Results shown from hCB_1R and hCB_2R are from the lab of Roche, mCB_1R and mCB_2R from the lab of Mauro Maccarrone. Results shown are obtained from three independent experiments performed in duplicate.

	hCB_2R		hCB_1R			mCB_2R		mCB_1R		
	pEC_{50} ± SEM	Emax ± SEM	pEC_{50} ± SEM	Emax ± SEM	CB_2R Selectivity*	pEC_{50} ± SEM	Emax ± SEM	pEC_{50} ± SEM	Emax	CB_2R Selectivity*
WIN55212-2	9.50 ± 0.09	98 ± 1	7.86 ± 0.23	107 ± 1	44	8.37 ± 0.23	97	8.04 ± 0.36	88	2
CP55940	10.33 ± 0.09	98 ± 1	9.73 ± 0.10	100 ± 3	4	10.17 ± 0.3	100	11.22 ± 0.1	100	0.1
Δ^9-THC	ND	ND	ND	ND	ND	ND	-	ND	-	ND
HU210	9.45 ± 0.27	94 ± 0.1	9.58 ± 0.24	104 ± 3	1	8.18 ± 0.45	68	10.03 ± 0.38	65	0.01
SR141716A	6.83 ± 0.06	-120 ± 17	8.19 ± 0.09	-118± 5	0.04	7.67 ± 0.67	-39	10.52 ± 0.44	-100	0.001
AM251	6.75 ± 0.03	-175 ± 19	8.30 ± 0.04	-124 ± 9	0.03	5.44 ± 0.23	-29	<5	-42	3
JWH015	8.52 ± 0.05	95 ± 1	<5	28 ± 1	3311	7.64 ± 0.24	93	<5	29	437
JWH133	8.38 ± 0.09	98 ± 1	<5	37 ± 6	2399	7.75 ± 0.33	95	<5	11	562
HU308	8.53 ± 0.06	98 ± 1	<5	18 ± 4	3388	8.43 ± 0.47	56	<5	10	2692
Gp-1a	7.52 ± 0.14	-85 ± 10	<5	19 ± 5	333	7.25 ± 0.32	-25	5.86 ± 0.42	-28	25
HU910	8.41 ± 0.13	98 ± 1	<5	28 ± 8	2570	8.98 ± 0.38	67	<5	36	9550
(rac)-AM1241	8.49± 0.08	67 ± 3	<5	n.a.	3103	7.03 ± 0.26	-6	<5	21	107
(R)-AM1241	8.76 ± 0.18	65 ± 2	6.17 ± 0.12	54 ± 7	391	6.17 ± 0.23	-22	5.08 ± 0.16	32	12
(S)-AM1241	7.40 ± 0.11	78 ± 1	<5	16 ± 2	250	7.6 ± 0.3	35	5.43 ± 0.42	16	148
AM630	7.56 ± 0.05	-152 ± 13	<5	28 ± 5	363	7.93 ± 0.3	-65	<5	13	851
SR144528	7.67 ± 0.18	-151 ± 10	<5	n.a.	468	8.25 ± 0.29	-100	5.54 ± 0.16	-39	513
Anandamide	5.93 ± 0.29	87± 7	<5	39 ± 3	8	6.82 ± 0.51	48	5.14 ± 0.21	69	48
2-AG	6.82 ± 0.05	94± 1	<5	n.a.	67	7.15 ± 0.4	53	5.16 ± 0.3	40	98

*CB_2R selectivity was calculated as follows: 10^(pEC50 CB_2R-pEC50 CB_1R)

The effect of agonists is normalized to the effect of 10 µM CP55940

The potency of antagonists/inverse agonists is determined in presence of the EC80 of CP55940

Effect at 10 µM

n.a.: not active

Supplementary Table 5. Functional potency, efficacy and selectivity in the β-AR recruitment assay. Results shown are obtained from three independent experiments performed in duplicate.

	hCB$_2$R		hCB$_1$R		CB$_2$R Selectivity*	mCB$_2$R	
	pEC$_{50}$ ± SEM	Emax ± SEM	pEC$_{50}$ ± SEM	Emax ± SEM		pEC$_{50}$ ± SEM	Emax ± SEM
WIN55212-2	7.93 ± 0.42	55 ± 5	6.74 ± 0.21	89 ± 2	15	ND	ND
CP55940	8.39 ± 0.21	80 ± 5	7.96 ± 0.2	94 ± 2	3	8.02 ± 0.27	96 ± 1
Δ^9-THC	-	31 ± 3#	6.29 ± 0.03	35 ± 3	-	ND	ND
HU210	ND	ND	ND	ND	ND	ND	ND
SR141716A	5.61 ± 0.32	-89 ± 3	8.25 ± 0.04	-104 ± 1	0.002	ND	ND
AM251	5.75 ± 0.34	-104 ± 8	8.62 ± 0.1	-105 ± 1	0.001	ND	ND
JWH015	7.53 ± 0.31	70 ± 15	5.82 ± 0.03	18 ± 2	51	ND	ND
JWH133	7.81 ± 0.22	62 ± 11	5.37 ± 0.05	40 ± 7	275	6.80 ± 0.17	63 ± 7
HU308	7.45 ± 0.07	57 ± 10	<5	5.0 ± 1	282	6.47 ± 0.13	69 ± 3
Gp-1a	5.86 ± 0.16	-147 ± 11	<5	-83 ± 7	13	ND	ND
HU910	7.75 ± 0.03	48 ± 6	5.31 ± 0.06	-97 ± 4	274	6.78 ± 0.16	66 ± 1
(rac)-AM1241	<5	14 ± 2	<5	6 ± 5	1	ND	ND
(R)-AM1241	7.52 ± 0.43	29 ± 4	5.59 ± 0.13	26 ± 2	86	ND	ND
(S)-AM1241	6.54 ± 0.44	29 ± 8	<5	2 ± 1	35	ND	ND
AM630	6.15 ± 0.08	-124 ± 6	5.56 ± 0.1	-120 ± 25	4	ND	ND
SR144528	7.47 ± 0.27	-111 ± 3	4.97 ± 0.13	-82 ± 5	316	ND	ND
Anandamide	6.21 ± 0.63	38 ± 3	-	36 ± 13#	-	ND	ND
2-AG	5.70 ± 0.21	80 ± 21	-	51 ± 21#	-	ND	ND

*CB$_2$R selectivity was calculated as follows: 10^(pEC50 CB$_2$R-pEC50 CB$_1$R)

The effect of agonists is normalized to the effect of 10 µM CP55940

The potency of antagonists/inverse agonists is determined in presence of the EC$_{80}$ of CP55940

Negative values represent inhibition of the EC$_{80}$ of CP55940 (>-100, indication of inverse agonism)

Effect at 10 µM

Emax at 10 µM treatment, no plateau observed

n.a.: not active

Supplementary Table 6. Functional potency, efficacy and selectivity in the pERK assay. Results shown are obtained from three independent experiments performed in duplicate.

	hCB_2R		hCB_1R		CB_2R Selectivity*
	pEC_{50} ± SEM	Emax ± SEM	pEC_{50} ± SEM	Emax ± SEM	
WIN55212-2	8.34 ± 0.36	92 ± 8	6.41 ± 0.11	101 ± 7	85
CP55940	7.96 ± 0.14	102 ± 2	7.98 ± 0.07	96 ± 8	1
Δ^9-THC	6.7 ± 0.56	41± 9	6.99 ± 0.26	55 ± 4	0.5
HU210	7.25 ± 0.07	105 ± 10	7.83 ± 0.11	96 ± 7	0.3
SR141716A	4.13 ± 0.06	-67 ± 10	6.85 ± 0.14	-110 ± 7	0.002
AM251	4.72 ± 0.06	-90 ± 3	7.09 ± 0.13	-116 ± 10	0.004
JWH015	ND	ND	ND	ND	-
JWH133	6.19 ± 0.07	96 ± 13	<4	10 ± 3	155
HU308	6.86 ± 0.11	93 ± 2	<4	18 ± 4	724
Gp-1a	n.a.	5 ± 6	n.a.	2 ± 3	1
HU910	5.50 ± 0.10	104 ± 9	<4	12 ± 4	32
(rac)-AM1241	7.6 ± 0.88	32 ± 12	5.07 ± 0.05	86 ± 3	339
(R)-AM1241	7.84 ± 0.29	75 ± 9	5.66 ± 0.19	78 ± 12	151
(S)-AM1241	6.92 ± 0.22	60 ± 11	<4	17 ± 3	832
AM630	5.09 ± 0.06	-92 ± 4	<4	-40 ± 2	12
SR144528	5.87 ± 0.07	-99 ± 3	<4	-21 ± 2	74
Anandamide	5.41 ± 0.35	62 ± 2	5.00 ± 0.26	85 ± 8	3
2-AG	5.41 ± 0.25	99 ± 11	5.21 ± 0.16	111 ± 10	2

*CB_2R selectivity was calculated as follows: 10^(pEC50 CB_2R-pEC50 CB_1R)

The effect of agonists is normalized to the effect of 10 µM CP55940

The potency of antagonists/inverse agonists is determined in presence of the EC80 of CP55940

Effect at 10 µM

n.a.: not active

Supplementary Table 7. Functional potency, efficacy and selectivity in the GIRK assay. Results shown are obtained from three independent experiments performed in duplicate.

	hCB_2R		hCB_1R		CB_2R Selectivity*
	pEC_{50} ± SEM	Emax ± SEM	pEC_{50} ± SEM	Emax ± SEM	
WIN55212-2	7.98 ± 0.17	87 ± 3	6.79 ± 0.09	98 ± 6	15
CP55940	7.98 ± 0.07	100	7.86 ± 0.05	100	1
Δ^9-THC	<5	<u>10 ± 4</u>	6.67 ± 0.2	62 ± 6	0.02
HU210	7.24 ± 0.16	105 ± 4	7.28 ± 0.17	97 ± 8	1
SR141716A	5.24 ± 0.13	-	7.13 ± 0.04	-	0.01
AM251	5.11 ± 0.12	-	7.15 ± 0.09	-	0.01
JWH015	7.32 ± 0.14	76 ± 3	<5	<u>18 ± 4</u>	209
JWH133	<5	<u>31 ± 12</u>	<5	<u>5 ± 1</u>	1
HU308	6.58 ± 0.13	82 ± 6	<5	<u>15 ± 10</u>	38
Gp-1a	5.99 ± 0.08	-	5.26 ± 0.1	-	5
HU910	5.87 ± 0.13	74 ± 2	<5	<u>7 ± 5</u>	7
(rac)-AM1241	5.23 ± 0.21	59 ± 14	<5	<u>36 ± 4</u>	2
(R)-AM1241	<5	<u>47 ± 13</u>	5.00 ± 0.1	51 ± 3	1
(S)-AM1241	5.6 ± 0.14	68 ± 13	<5	<u>19 ± 3</u>	4
AM630	5.82 ± 0.08	-	5.08 ± 0.07	-	5
SR144528	5.21 ± 0.14	-	<5	<u>9 ± 4</u>	2
Anandamide	5.29 ± 0.21	51 ± 5	5.81 ± 0.3	91 ± 14	0.3
2-AG	6.51 ± 0.08	100 ± 2	6.35 ± 0.21	100 ± 11	1

*CB_2R selectivity was calculated as follows: 10^(pEC50 CB_2R-pEC50 CB_1R)

The effect of agonists is normalized to the effect of 10 µM CP55940

The potency of antagonists/inverse agonists is determined in presence of the EC80 of CP55940

<u>Effect at 10 µM</u>

Supplementary Table 8. Transduction ratios (logR) of the cannabinoid ligand library on hCB$_2$R. Normalized dose-response data determined in three independent experiments performed in duplicate (with the exception of AEA (N=2) and 2-AG (N=1) on cAMP) on different assays were analyzed using the operational model to determine LogR values. ΔlogR values were calculated for each ligand using CP55940 as the reference ligand with equation 1, the standard errors of the ΔlogR values with equation 2, and the relative effectiveness (RE) with equation 3 (See Supplementary Methods). Statistics performed was one-way ordinary ANOVA.

Ligand	B-AR			GTPγS			pERK			GIRK			cAMP		
	LogR	ΔLogR	RE	Log R	ΔLogR	RE	Log R	ΔLogR	RE	LogR	ΔLogR	RE	LogR	ΔLogR	RE
WIN55212-2	8.10 ± 0.10	-0.4 ± 0.11	0.40	7.49 ± 0.39	-0.8 ± 0.45	0.16	9.29 ± 0.17	0.26 ± 0.24	1.81	7.62 ± 0.09	-0.25 ± 0.10	0.57	9.40 ± 0.07	-1.31 ± 0.10	0.049*
JWH133	8.02 ± 0.11	-0.48 ± 0.12	0.33	6.27 ± 0.31	-2.01 ± 0.39	0.01*	7.07 ± 0.17	-1.96 ± 0.24	0.01*	5.98 ± 0.41	-1.89 ± 0.41	0.01*	8.28 ± 0.05	-2.44 ± 0.09	0.004*
HU910	7.72 ± 0.11	-0.78 ± 0.12	0.16	6.82 ± 0.18	-1.46 ± 0.29	0.03	6.94 ± 0.2	-2.1 ± 0.26	0.01*	5.90 ± 0.17	-1.96 ± 0.18	0.01*	8.37 ± 0.07	-2.35 ± 0.09	0.005*
CP55940	8.5 ± 0.05	0 ± 0.07	1.00	8.28 ± 0.23	0 ± 0.33	1.00	9.03 ± 0.17	0 ± 0.24	1.00	7.863± 0.05	0 ± 0.07	1.00	10.17 ± 0.07	0 ± 0.09	1.00
HU308	7.72 ± 0.12	-0.78 ± 0.13	0.17	6.95 ± 0.17	-1.33 ± 0.29	0.05	7.55 ± 0.2	-1.48 ± 0.26	0.03*	6.281± 0.13	-1.58 ± 0.14	0.03*	8.49 ± 0.05	-2.22 ± 0.08	0.006*
(rac)-AM1241	7.95 ± 0.44	-0.55 ± 0.44	0.28	5.9 ± 0.35	-2.38 ± 0.42	0.004*	8.67 ± 0.66	-0.37 ± 0.68	0.43	5.52 ± 0.19	-2.35 ± 0.20	0.004*	ND	ND	ND
(S)-AM1241	5.97 ± 0.28	-2.54 ± 0.28	0.003*	6.9 ± 0.43	-1.38 ± 0.49	0.04	7.54 ± 0.28	-1.49 ± 0.33	0.03*	5.70 ± 0.16	-2.17 ± 0.17	0.01*	ND	ND	ND
(R)-AM1241	7.21 ± 0.26	-1.30 ± 0.27	0.05*	7.3 ± 0.41	-0.99 ± 0.47	0.10	8.12 ± 0.22	-0.91 ± 0.28	0.12	5.48 ± 0.24	-2.38 ± 0.25	0.004*	ND	ND	ND
JWH015	7.62 ± 0.11	-0.88 ± 0.13	0.13*	5.96 ± 0.33	-2.32 ± 0.4	0.005*	ND	ND	ND	6.78 ± 0.09	-1.09 ± 0.11	0.08*	8.45 ± 0.06	-2.26 ± 0.09	0.006*
AEA	5.54 ± 0.22	-2.96 ± 0.23	0.001*	4.54 ± 0.53	-3.74 ± 0.58	0.0002*	6.03 ± 0.27	-3 ± 0.32	0.001*	4.53 ± 0.28	-3.33 ± 0.29	0.005*	5.96 ± 0.17	-4.76 ± 0.18	0.00002*
2-AG	5.9 ± 0.14	-2.6 ± 0.15	0.003*	4.63 ± 0.36	-3.65 ± 0.43	0.0002*	6.37 ± 0.2	-2.67 ± 0.26	0.002*	6.40 ± 0.11	-1.47 ± 0.12	0.03*	6.99 ± 0.16	-3.72 ± 0.18	0.0002#
Δ^9-THC	5.5 ± 0.28	-3.0 ± 0.28	0.001*	4.51 ± 0.73	-3.78 ± 0.77	0.0002*	7.89 ± 0.48	-1.15 ± 0.51	0.07	ND	ND	ND	ND	ND	ND
HU210	ND	ND	ND	ND	ND	ND	8.22 ± 0.17	-0.82 ± 0.24	0.15	7.19 ± 0.10	-0.67 ± 0.11	0.21*	ND	ND	ND

ND = not determined
*** P-value < 0.05**
# No statistical analysis performed

Supplementary Table 9. Transduction ratios (logR) of the cannabinoid ligand library on mCB_2R. Normalized dose-response data determined in three independent experiments, performed in duplicate, on different assays were analyzed using the operational model to determine LogR values. ΔlogR values were calculated for each ligand using CP55940 as the reference ligand with equation 1, the standard errors of the ΔlogR values with equation 2, and the relative effectiveness (RE) with equation 3 (supplemental information). Statistics performed was one-way ordinary ANOVA.

Ligand	B-AR			GTPγS			cAMP		
	LogR	ΔLogR	RE	LogR	ΔLogR	RE	LogR	ΔLogR	RE
**CP55940**	8.0 ± 0.06	0 ± 0.08	1.00	7.82 ± 0.05	0 ± 0.07	1.00	10.52 ± 0.14	0 ± 0.20	1.00
**HU308**	6.22 ± 0.10	-1.77 ± 0.12	0.02*	6.77 ± 0.05	-1.04 ± 0.07	0.09*	8.07 ± 0.22	-2.46 ± 0.26	0.004*
**HU910**	6.57 ± 0.11	-1.41 ± 0.12	0.04*	7.15 ± 0.07	-0.67 ± 0.08	0.22*	8.48 ± 0.18	-2.04 ± 0.23	0.009*
**JWH133**	6.57 ± 0.12	-1.42 ± 0.13	0.04*	7.07 ± 0.05	-0.75 ± 0.07	0.18*	8.07 ± 0.09	-2.45 ± 0.17	0.004*

*** P-value < 0.05**

Supplementary Table 10. ΔΔlogR values and bias factor for the reference library between pathways on hCB_2R. Normalized dose-response data determined in at least three independent experiments performed in duplicate (with the exception of AEA (N=2) and 2-AG (N=1) on cAMP) on different assays were analyzed using the operational model to determine LogR values (see for a step-by-step explanation the Supplemental Methods). ΔΔlogR values are calculated using the ΔlogR values (Table S8) using equation 4, the standard errors of the ΔΔlogR values with equation 5, and the bias factor (BF) using equation 6. Statistics performed was one-way ordinary ANOVA.

Ligand	β-AR-GTPγS	β-AR-pERK	β-AR-GIRK	β-AR-cAMP	pERK-GTPγS	pERK-GIRK	pERK-cAMP	GIRK-GTPγS	GIRK-cAMP	GTPγS-cAMP
	ΔΔlogR (BF)	ΔΔlogR (BF)	ΔΔlogR (BF)	ΔΔlogR (BF)	ΔΔlogR (BF)	ΔΔlogR (BF)	ΔΔlogR (BF)	ΔΔlogR (BF)	ΔΔlogR (BF)	ΔΔlogR (BF)
**WIN55212-2**	0.4 ± 0.47 (2.49)	-0.66 ± 0.27 (0.22)	-0.15 ± 0.15 (0.70)	0.91 ± 0.15 (8.2)*	1.05 ± 0.51 (11.30)	0.51 ± 0.26 (3.24)	1.57 ± 0.26 (37.24)*	0.55 ± 0.46 (3.55)	1.07 ± 0.14 (11.69)*	0.52 ± 0.46 (3.30)
**JWH133**	1.53 ± 0.41 (33.81)	1.48 ± 0.27 (30.41)	1.41 ± 0.43 (25.53)*	1.96 ± 0.15 (90.36)*	0.05 ± 0.46 (1.11)	-0.08 ± 0.48 (0.84)	0.47 ± 0.26 (2.97)	0.12 ± 0.57 (1.32)	0.55 ± 0.42 (3.54)	0.43 ± 0.40 (2.67)
**HU910**	0.67 ± 0.32 (4.71)	1.31 ± 0.29 (20.56)	1.18 ± 0.22 (15.07)	1.57 ± 0.15 (36.90)*	-0.64 ± 0.39 (0.23)	-0.13 ± 0.32 (0.73)	0.25 ± 0.28 (1.77)	-0.51 ± 0.34 (0.31)	0.38 ± 0.20 (2.42)	0.89 ± 0.31 (7.73)
**CP55940**	0 ± 0.34 (1.00)	0 ± 0.25 (1.00)	0 ± 0.1 (1.00)	0 ± 0.12 (1.00)*	0 ± 0.41 (1.00)	0 ± 0.25 (1.00)	0 ± 0.26 (1.00)	0 ± 0.34 (1.00)	0 ± 0.12 (1.00)	0 ± 0.34 (1.00)
**HU308**	0.55 ± 0.32 (3.54)	0.71 ± 0.29 (5.08)	0.80 ± 0.19 (6.37)	1.43 ± 0.16 (27.16)*	-0.16 ± 0.39 (0.70)	0.1 ± 0.3 (1.25)	0.73 ± 0.27 (5.42)	-0.26 ± 0.32 (0.56)	0.64 ± 0.17 (4.33)	0.89 ± 0.30 (7.78)
**(rac)-AM1241**	1.83 ± 0.61 (68.30)	-0.19 ± 0.81 (0.65)	1.8 ± 0.49 (62.37)*	ND	2.01 ± 0.8 (103.28)	1.98 ± 0.71 (95.72)*	ND	0.03 ± 0.46 (1.08)	ND	ND
**(S)-AM1241**	-1.16 ± 0.57 (0.07)	-1.05 ± 0.44 (0.09)	-0.37 ± 0.33 (0.43)	ND	-0.11 ± 0.59 (0.77)	0.68 ± 0.37 (4.72)	ND	-0.79 ± 0.52 (0.16)	ND	ND
**(R)-AM1241**	-0.31 ± 0.54 (0.49)	-0.38 ± 0.39 (0.41)	1.08 ± 0.36 (12.13)	ND	0.07 ± 0.55 (1.18)	1.47 ± 0.37 (29.32)	ND	-1.39 ± 0.53 (0.04)	ND	ND
**JWH015**	1.44 ± 0.42 (27.80)	ND	0.21 ± 0.17 (1.61)	1.38 ± 0.15 (24.10)*	ND	ND	ND	1.24 ± 0.42 (17.26)	1.18 ± 0.14 (14.96)*	-0.06 ± 0.41 (0.87)
**AEA**	0.79 ± 0.62 (6.10)	0.04 ± 0.39 (1.11)	0.38 ± 0.37 (2.37)	1.80 ± 0.29 (62.52)*	0.74 ± 0.66 (5.50)	0.33 ± 0.43 (2.14)	1.75 ± 0.37 (56.36)*	0.41 ± 0.65 (2.57)	1.42 ± 0.34 (26.36)*	1.01 ± 0.41 (10.26)
**2-AG**	1.05 ± 0.45 (11.27)	0.07 ± 0.3 (1.16)	-1.14 ± 0.19 (0.07)	1.12 ± 0.23 (13.03)#	0.99 ± 0.5 (9.68)	-1.2 ± 0.29 (0.06)	1.05 ± 0.31 (11.19)#	2.19 ± 0.44 (154.53)*	2.25 ± 0.21 (178.65)#	0.06 ± 0.46 (1.16)#
**Δ^9-THC**	0.77 ± 0.82 (5.90)	-1.86 ± 0.58 (0.01)*	ND	ND	2.63 ± 0.92 (426.58)*	ND	ND	ND	ND	ND
**HU210**	ND	ND	ND	ND	ND	-0.15 ± 0.27 (0.71)	ND	ND	ND	ND

ND = not determined
* P-value < 0.05
# No statistical analysis performed

Supplementary Table 11. ΔΔlogR values and bias factor for the cannabinoid ligand library between pathways on mCB_2R. Normalized dose-response data determined in three independent experiments, performed in duplicate, on different assays were analyzed using the operational model to determine LogR values (see for a step-by-step explanation the Supplemental Methods). ΔΔlogR values are calculated using the ΔlogR values (Table S8) using equation 4, the standard errors of the ΔΔlogR values with equation 5, and the bias factor (BF) using equation 6. Statistics performed was one-way ordinary ANOVA.

Ligand	β-AR-GTPγS	β-AR-cAMP	GTPγS-cAMP
	ΔΔlogR (BF)	ΔΔlogR (BF)	ΔΔlogR (BF)
CP55940	0 ± 0.10 (1)	0 ± 0.21 (1)	0 ± 0.21 (1)
HU308	-0.72 ± 0.13 (0.19)*	0.69 ± 0.28 (4.9)	1.41 ± 0.27 (25.8)*
HU910	-0.75 ± 0.15 (0.18)*	0.63 ± 0.26 (4.2)	1.37 ± 0.24 (23.6)*
JWH133	-0.67 ± 0.15 (0.21)*	1.04 ± 0.21 (10.9)*	1.71 ± 0.18 (50.9)*
*** P-value < 0.05**			

Supplementary Table 12. Off-target activity on ECS enzymes. None of the cannabinoid ligands showed covalent interaction with a panel of serine hydrolases in a competitive activity-based protein profiling assay,[1] but some were tested additionally on the particular ECS enzymes to double check their inactivity on these enzymes. Data from single-point experiments are obtained from two independent experiments performed in duplicate. Data from full dose-response experiments are obtained from three independent experiments performed in duplicate.

Compound	ABHD12 IC_{50} in µM (95% CI) or % inhibition at 10 5 µM)	FAAH (% inhibition at 5 µM)	NAPE-PLD (% inhibition at 10 µM ± SD)	DAGL (% inhibition at 10 µM ± SD)	MAGL (% inhibition at 10 µM ± SD)
**WIN55212-2**	<50	<25	8 ± 6	-9 ± 12	3 ± 18
**CP55940**	ND	ND	10 ± 8	20 ± 16	5 ± 16
**Δ^9-THC**	ND	ND	0 ± 12	-9 ± 12	7 ± 17
**HU210**	ND	ND	ND	ND	ND
**SR141716A**	6.1 (4.1-9.1)	<25	0 ± 7	20 ± 10	4 ± 18
**AM251**	1.6 (1.3-2.0)	<50	10 ± 12	17 ± 6	0 ± 16
**JWH015**	ND	ND	-4 ± 12	-8 ± 5	0 ± 17
**JWH133**	ND	ND	3 ± 4	30 ± 7	-20 ± 5
**HU308**	<25	<25	2 ± 7	42 ± 3	7 ± 13
**Gp-1a**	0.8 (0.58-1.1)	<50	3 ± 5	ND	-1 ± 15
**HU910**	<25	<25	8 ± 13	36 ± 1	20 ± 12
**(rac)-AM1241**	ND	ND	29 ± 11	3 ± 10	-2 ± 17
**(R)-AM1241**	<25	<25	21 ± 5	10 ± 12	-2 ± 18
**(S)-AM1241**	<25	<25	33 ± 7	9 ± 9	-4 ± 18
**AM630**	<25	ND	-4 ± 11	6 ± 12	4 ± 16
**SR144528**	<25	ND	8 ± 10	-28 ± 11	3 ± 16
**Anandamide**	ND	ND	17 ± 9	32 ± 22	-2 ± 19
**2-AG**	ND	ND	-1 ± 12	-12 ± 16	3 ± 15

Supplementary Table 13. Off-target activity data on GPR55. Ability of the ligands to signal through GPR55 was measured by determination of βArrestin recruitment by the receptor after addition of the ligands. Data from single-point experiments are obtained from two independent experiments performed in duplicate. Data from full dose-response experiments are obtained from three independent experiments performed in duplicate.

Compound	GPR55	
	pEC50 (± SEM)	Emax ± SEM (%) or % effect at 10 μM
WIN55212-2	<5	5
CP55940	<5	8 ± 18
Δ^9-THC	ND	ND
HU210	ND	ND
SR141716A	ND	ND
AM251	5.49 (± 0.09)	82 ± 9
JWH015	<5	2
JWH133	<5	-2 ± 3
HU308	<5	-6 ± 5
Gp-1a	<5	-10
HU910	<5	-10 ± 3
(rac)-AM1241	ND	ND
(R)-AM1241	ND	ND
(S)-AM1241	ND	ND
AM630	<5	-19
SR144528	<5	-14
Anandamide	<5	-9
2-AG	ND	ND

Supplementary Table 14. AEA reuptake inhibition data. Data from single-point experiments are obtained from two independent experiments performed in duplicate. Data from full dose-response experiments are obtained from three independent experiments performed in duplicate.

	HaCaT cells		U937 cells		HMC-1 cells
	IC_{50}, µM (95% CI)	Efficacy (% inhibition)	IC_{50}, µM (95% CI)	Efficacy (% inhibition)	IC_{50}, µM (95% CI)
**WIN55212-2**	>5	24*	4.65 (3.28 – 6.59)	52	> 5
**CP55940**	>10	13*	ND	ND	ND
**Δ^9-THC**	ND	ND	ND	ND	ND
**HU210**	>5	44*	ND	ND	ND
**SR141716A**	2.90 (0.61-13.8)	80	1.91 (0.88 – 4.15)	57	3.5 (1.4-5.1)
**AM251**	1.40 (0.23-8.22)	54	1.05 (ND)	67	> 5
**JWH015**	>5	64*	ND	ND	ND
**JWH133**	>10	39*	ND	ND	ND
**HU308**	2.21(1.10-4.50)	69	1.81 (1.25 – 2.64)	62	> 5
**Gp-1a**	3.27 ND	90	1.26 (ND)	64	> 5
**HU910**	>10	38*	1.56 (1.20 – 2.02)	70	> 5
**(rac)-AM1241**	4.01 ND	80	ND	ND	ND
**(R)-AM1241**	>5	25*	4.81 (3.83 – 6.03)	77	> 5
**(S)-AM1241**	4.49 (1.56-12.9)	90	3.70 (2.78 – 4.92)	97	> 5
**AM630**	4.18 (0.50-35.3)	65	> 10	23*	> 10
**SR144528**	>10	20*	> 10	30*	> 10
**Anandamide**	0.27 (0.15-0.497)	86	ND	ND	ND
**2-AG**	>5	60*	ND	ND	ND

*Effect at 10 µM

Supplementary Table 15. TRP channel data. Data from single-point experiments are obtained from two independent experiments performed in duplicate. Data from full dose-response experiments are obtained from three independent experiments performed in duplicate.

	**Rat recombinant TRPA1**			**Human recombinant TRPV1**			**Rat recombinant TRPM8**	**Rat recombinant TRPV2**			**Rat recombinant TRPV3**			**Rat recombinant TRPV4**		
	**Efficacy (%AITC 100µM)**	**Potency EC50 (µM)**	**IC50 (µM) for antagonism or desensitization (* AITC 100µM)**	**Efficacy (% ionomycin 4µM)**	**Potency EC50 (µM)**	**IC50 (µM) for antagonism or desensitization (*capsaicin, 0.1µM, or *capsaicin, 10 nM*)**	**IC50 (µM, unless otherwise stated) for antagonism of icilin, 0.25µM, or *icilin, 0.1µM*)**	**Efficacy (% ionomycin 4µM)**	**Potency EC_{50} µM**	**IC_{50} (µM) for antagonism or desensitization (*LPC, 3µM)**	**Efficacy (% ionomycin 4µM)**	**Potency EC_{50} µM**	**IC_{50} (µM) for antagonism or desensitization (*thymol, 100µM)**	**Efficacy (% ionomycin 4µM)**	**Potency EC_{50} µM**	**IC_{50} (µM) for desensitization (* 4αPDD, 1µM)**
**WIN55212-2**	72.3 ± 0.9	2.3 ± 0.1	6.4 ± 0.6	44.4 ± 0.9	19.2 ± 1.3	35.8 ± 2.2	72.9 ± 4.5 *19.0 ± 2.2*	17.0 ± 0.5	14.8 ± 1.4	62.0 ± 7.2	22.9 ± 0.6	6.5 ± 0.9	> 100	< 10	n.a.	16.1 ± 1.7
**SR141716A**	67.3 ± 1.2	1.9 ± 0.1	12.5 ± 2.2	17.5 ± 1.5	12.0 ± 0.6	33.7 ± 6.6 *11.5 ± 1.5*	*41.0 ± 10.9 nM*	< 10	n.a.	> 100	38.9 ± 2.1	0.85 ± 0.15	3.4 ± 0.4	< 10	n.a.	2.0 ± 0.1
**AM251**	44.4 ± 0.7	0.86 ± 0.06	17.1 ± 2.2	< 10	n.a.	> 50	18.4 ± 3.5 *1.5 ± 0.1*	< 10	n.a.	18.4 ± 3.5	25.9 ± 1.3	0.6 ± 0.1	> 50	< 10	n.a.	1.2 ± 0.1
**HU308**	43.1 ± 2.2	18.5 ± 3.9	> 100	< 10	n.a.	69.0 ± 5.7	> 100	< 10	n.a.	> 100	< 10	n.a.	> 100	< 10	n.a.	> 100
**Gp-1a**	83.6 ± 0.9	2.1 ± 0.1	10.4 ± 1.4	< 10	n.a.	3.0 ± 0.1	> 50	< 10	n.a.	11.9 ± 0.7	< 10	n.a.	22.6 ± 3.9	< 10	n.a.	2.2 ± 0.1
**HU910**	33.1 ± 0.1	53.1 ± 1.1	> 100	< 10	n.a.	> 100	> 100	< 10	n.a.	> 100	31.3 ± 2.2	0.12 ± 0.05	12.9 ± 4.2	< 10	n.a.	> 100
**(R)-AM1241**	19.8 ± 1.3	19.5 ± 5.8	> 50	10.7 ± 1.5	> 50	> 50	> 50	14.5 ± 0.3	12.0 ± 3.1	35.5 ± 1.5	12.9 ± 0.1	10.0 ± 0.1	> 50	< 10	n.a.	8.7 ± 0.5
**(S)-AM1241**	47.5 ± 0.8	5.8 ± 0.4	40.9 ± 5.9	12.0 ± 1.2	> 50	> 50	> 50	11.6 ± 0.1	5.0 ± 0.1	20.6 ± 3.1	16.2 ± 0.1	9.0 ± 0.1	> 50	< 10	n.a.	8.6 ± 0.3
**AM630**	118.0 ± 2.0	1.9 ± 0.2	3.7 ± 0.5	< 10	n.a.	> 100	4.3 ± 0.3 *1.9 ± 0.1*	< 10	n.a.	35.6 ± 1.4	< 10	n.a.	> 100	< 10	n.a.	3.2 ± 0.1
**SR144528**	43.8 ± 1.4	8.9 ± 1.2	> 100	< 10	n.a.	> 100	2.9 ± 1.3	< 10	n.a.	> 50	< 10	n.a.	> 100	< 10	n.a.	> 100
**JWH133**	76.8 ± 3.8	8.5 ± 2.3	20.0 ± 3.2	24.6 ± 0.4	8.2 ± 0.7	77.7 ± 3.0	48.4 ± 3.5	< 10	n.a.	> 100	< 10	n.a.	80.6 ± 1.4	13.6 ± 0.8	12.0 ± 3.0	> 100

*5 min pre-incubation with indicated compound

Supplementary Table 16. Summary table of off-target activity per compound. Data shown is % efficacy at 10 µM, IC_{50} ± SEM or IC_{50} (95% confidence interval). The ligands are organized according to their amount of off-targets. CEREP panel data is obtained from two independent experiments performed in duplicate.

Ligand	Trp channels	ECS proteins or GPR55	AEA reuptake inhibition	CEREP panel	Total
**CP55940**	ND	-	ND (2/3)	A_3 receptor (98%)<br>α1A (57%)<br>AT1 (63%)<br>D1 (77%)<br>FP (100%)<br>GR (92%)<br>$5\text{-}HT_{1A}$, $5\text{-}HT_{2A}$, $5\text{-}HT_{2B}$ receptor (92, 95, 96%)<br>Kappa (KOP) (74%)<br>M4 (55%)<br>mu (MOP) (57%)<br>Na^+ channel (71%)<br>Norepinephrine transporter (89%)<br>PPARγ (85%)<br>Sigma (60%)<br>Sst4 (63%)	17
**AM251**	TRPA1 (0.86 ± 0.06 µM)<br>TRPV3 (0.6 ± 0.1 µM)	ABHD12 (1.6 µM (1.3-2.0))<br>GPR55 (82%)	U937 cells (67%)<br>HaCaT cells (54%)	A_3 receptor (93%)<br>D1 (58%)<br>FP (94%)<br>GR (84%)<br>Kappa (KOP) (94%)<br>Mu (MOP) (93%)<br>Na^+ channel (57%)<br>Norepinephrine transporter (56%)<br>PPARγ (55%)<br>Sigma (59%)<br>Sst4 (51%)	15
**WIN55212-2**	TRPA1 (72 ± 1%)	-	U937 cells (52%)	A_3 receptor (84%)<br>Ca^{2+} channel (55%)<br>FP (81%)<br>GR (63%)<br>$5\text{-}HT_{2B}$ receptor (70%)<br>kappa (KOP) (79%)<br>MT3 (ML2) (69%)<br>Na^+ channel (68%)<br>Sigma (53%)<br>Sst4 (64%)<br>COX2 (50%)	13
**Gp-1a**	TRPA1 (84 ± 1%)	ABDH12 (0.8 µM (0.58-1.1))	U937 cells (64%)<br>HaCaT cells (90%)	A_3 receptor (95%)<br>D1 (63%)<br>D2S (53%)<br>FP (88%)<br>GR (71%)<br>Kappa (KOP) (99%)<br>Mu (MOP) (102%)<br>Norepinephrine transporter (59%)	13

				PPARγ (86%)<br>Sigma (51%)	
**(S)-AM1241**	-	-	U937 cells (97%)<br>HaCaT cells (90%)	α1A (75%)<br>Ca^{2+} channel (90%)<br>H_1, H_3 receptor (78, 87%)<br>5-HT_{1A}, 5-HT_{2A}, 5-HT_{2B} receptor (74, 67, 93%)<br>Kappa (KOP) (87%)<br>M4 (74%)<br>Na^+ channel (87%)<br>Sigma (101%)<br>Sst4 (63%)	13
**AM630**	TRPA1 (118 ± 2%)	-	HaCaT cells (65%)	A_3 receptor (76%)<br>CB_1 receptor (66%) (confirmed with full dose-response)<br>Cl^- channel (87%)<br>FP (66%)<br>5-HT_{2A}, 5-HT_{2B} receptor (70, 65%)<br>Kappa (KOP) (57%)<br>PPARγ (99%)<br>COX2 (96%)	11
**SR141716A**	TRPA1 (67 ± 1%)<br>TRPM8 (41 ± 11 nM)<br>TRPV3 (0.85 ± 0.15 µM)	ABHD12 (6.1 µM (4.1-9.1))	U937 cells (57%)<br>HaCaT cells (80%)	Cl^- channel (83%)<br>MC3 (53%)<br>MT1 (ML1A) (62%)	8
**JWH015**	ND	-	HaCaT cells (64%)<br>ND (2/3)	A_3 receptor (93%)<br>5-HT_{2A}, 5-HT_{2B} receptor (56, 94%)<br>Kappa (KOP) (53%)<br>Na^+ channeld (50%)<br>PPARγ (67%)	7
**HU308**	-	-	U937 cells (62%)<br>HaCaT cells (69%)	AT1 (53%)<br>CCK1 (CCKA) (75%)<br>Dopamine transporter (71%)<br>NK2 (68%)	5
**HU910**	TRPV3 (0.12 ± 0.05 µM)	-	U937 cells (70%)	None of the 9 off-targets found in single-point screen (Table S15) were confirmed in full dose-response experiments	2
**SR144528**	-	-	-	A_3 receptor (72%)<br>PDE5 (71%)	2
**JWH133**	TRPA1 (77 ± 4%)	-	ND (2/3)	-	1
**Δ^9-THC**	ND	-	ND (3/3)	ND	ND
**HU-210**	ND	-	ND (2/3)	ND	ND
**(rac)-AM1241**	ND	-	HaCaT cells (80%)<br>ND (2/3)	ND	ND
**(R)-AM1241**	-	-	U937 cells (77%)	ND	ND
**Anandamide**	ND	-	HaCaT cells (86%)<br>ND (2/3)	ND	ND
**2-AG**	ND	-	HaCaT cells (60%)<br>ND (2/3)	ND	ND

ND: not determined, ND (2/3): not determined in two out of three cellines, ND (3/3): not determined in three out of three cellines
- No off-target activity in this protein family

Supplementary Table 17. *In vitro* ADME parameters. Hepatocyte and microsomal clearance was measured in single experiment, with multiple timepoints. Plasma protein binding and P-gp binding is measured in triplicates**.**

Ligand	CL microsomes [µL/min/mg protein]	CL hepatocytes [µL/min/mio cells]	Plasma protein binding [%free]	P-gp (ER[1])	CL microsomes [µL/min/mg protein]	CL hepatocytes [µL/min/mio cells]	Plasma protein binding [%free]	P-gp (ER)
	human				Mouse			
WIN55212	194	42	<0.3	1.5	745	568	1.0	1.4
CP55940	80	n.d.[2]	n.d.	n.d.	172	n.d.	n.d.	n.d.
THC	n.d.	n.d.	n.d.	n.d.	n.d.	n.d.	n.d.	n.d.
HU210	25	n.d.	n.d.	n.d.	33	n.d.	n.d.	n.d.
SR141716A	20	5.7	<0.1	1.4	109	65	<0.1	1.5
AM251	18	3.6	<0.1	1.3	70	53	<0.1	1.8
JWH015	41	16	<0.1	1.5	989	205	<0.1	1.5
JWH133	38	5.6	<0.1	n.d.	63	9.8	<0.1	1.7
HU308	n.d.	8.3	n.d.	6.2	n.d.	4.7	n.d.	7.3
Gp-1a	13	32	<0.1	1.0	134	78	<0.1	1.1
HU910	90	9.2	1.8	2.2	464	34	4.1	3.0
(Rac)-AM1241	111	n.d.	n.d.	n.d.	254	n.d.	n.d.	n.d.
(R)-AM1241	304	n.d.	n.d.	n.d.	314	n.d.	n.d.	n.d.
(S)-AM1241	152	37	0.5	2.7	640	251	1.2	1.4
AM630	82	16	<0.1	1.3	468	101	0.4	1.3
SR 144528	129	n.d.	<0.2	4.1	446	n.d.	n.d.	5.6
Anandamide	47	n.d.	n.d.	n.d.	222	n.d.	n.d.	n.d.
2-AG	13	n.d.	n.d.	n.d.	12.0	n.d.	n.d.	n.d.

1) ER: efflux ratio;2) n.d.: not determined;

Supplementary Table 18. Pharmacokinetic parameters following intravenous administration in mice. Sample size was 6 animals for each compound

Ligand	Dose mg/kg	C0 ng/mL	Cmax [mg/mL	AUClast h*ng/mL	AUCinf h*ng/mL	CL L/h/kg	T1/2 h	Vss L/kg	AUCinf/dose h*kg*ng/mL/mg
**(S)-AM1241**	1.9	413	316	269	276	6.9	1.4	9.3	145
**AM251**	1.5	1180	995	1250	1390	1.1	3.1	2.8	923
**AM630**	1.0	69.8	286	177	197	5.1	2.9	14.0	197
**Gp-1a**	2.0	351	435	658	825	2.4	3.5	9.9	412
**HU910**	1.0	55600	15400	5960	6060	0.17	7.4	0.26	6060
**JWH015**	1.1	438	262	163	174	6.3	2.6	10.1	158
**JWH133**	1.6	3870	2480	1650	1750	0.92	1.1	1.32	1090
**SR144528**	1.0	15800	3170	1430	1440	0.69	2.1	0.50	1440

Supplementary Table 19. Pharmacokinetic parameters following oral administration in mice and rat. Sample size was 6 animals for each compound

Compound	Species	Dose [mg/kg]	Tmax [h]	Cmax [ng/mL]	AUClast [h*ng/mL]	AUCinf [h*ng/mL]	T1/2 [h]	AUCinf/dose [h*kg*ng/mL/mg]
HU910	Mice	3.0	1.0	495	895	904	5.2	301
HU308	Mice	5.0	0.5	201	253	298	3.3	59.7
JWH133	Rat	6.0	1.1	2070	3330	34500	2.7	583

Supplementary Table 20. CEREP panel data. Data shown is the percentage of inhibition for binding assays and the percentage of inhibition for enzyme and cell-based assays at a test concentration of 10 µM. Data presented is obtained from two independent experiments performed in duplicate.

**Assay**	**Compound**											
**Short name**	**WIN55212-2**	**CP55940**	**SR141716A**	**AM251**	**JWH015**	**JWH133**	**HU308**	**Gp-1a**	**HU910**	**(S)-AM1241**	**AM630**	**SR144528**
Binding assays												
**A1 (h)**	10	21		27	10	-6	5	25	23	14	28	4
**A2A (h)**						4	25		50			
**A2B**			-7									
**A3 (h)**	84	98		93	93	-26	-1	95	62	42	76	72
**alpha 1A (h)**	15	57		6	16	-5	2	11	1	75	47	-1
**alpha 2A (h)**	0	23		30	1	-18	-6	30	7	10	-5	-5
**AR (h)**	8	-28	4	18	0			-8		1	-7	-12
**AT1 (h)**	29	63		36	21	7	53	20	37	1	17	33
**AT2 (h)**						0	42		13			
**B1**			-3									
**B2 (h)**						-2	-21		-39			
**BB**						0	3		18			
**beta 1 (h)**	6	14		25	7	-7	-11	30	-4	14	9	41
**beta 2 (h)**						-15	-30		-5		11	
**BLT1 (LTB4)**			14									
**BZD (central)**	4	-9		-79	-57	-22	-26	-10	0	-1	-12	-3
**BZD (peripheral)**						-1	12		-11			
**C5a**			2									
**Ca2+ channel (L, diltiazem site)**	55	-7		49	33			34		90	-11	10
**Ca2+ channel (verapamil site)**						1	3		7			
**CB1 (h)**						6	18		71		66	

**CCK1 (CCKA) (h)**						48	75		63		1	
**CCK2 (CCKB) (h)**						4	7		18			
**CCR1 (h)**						11	4		-2			
**Cl- channel (GABA-gated)**			83			2	30		49		87	
**CGRP (h)**						-13	-34		-57			
**CRF1**			-16									
**CXCR2 (IL-8B) (h)**						-11	3		69			
**CysLT1 (LTD4)**			-25									
**D1 (h)**	19	77		58	38	-4	3	63	26	31	29	24
**D2S (h)**						11	12		-11			
**D2S (h)**	-3	37		16	-13			53		37	13	-8
**D3 (h)**						6	35		24			
**D4.4 (h)**						10	-4		-13			
**D5 (h)**						-21	-3		8			
**delta2 (DOP) (h)**						-1	2		9			
**dopamine transporter (h)**						20	71		66			
**EP2 (h)**						-2	16		28			
**ER**			-17						6			
**ERalpha (h)**	5	12		-12	11			-21		-1	9	-15
**Eralpha**			-5									
**Erbeta**			-27									
**ETA (h)**						-6	1					
**ETB (h)**						4	48		-4			
**FP (h)**	81	100		94	26			88		12	66	28
**GABA**						-3	-3		14			
**GAL1 (h)**						-22	-22		10			
**GAL2 (h)**						-7	-6		-1			
**glycine (strychnine-insensitive)**	-14	8		-13	4			-9		-15	-2	2

**ghrelin (GHS)**			3									
**glucagon**			-13									
**GnRH (LH-RH)**			-8									
**GR (h)**	63	94	43	84	36	7	4	71	4	10	38	9
**H1 (h)**	20	49		42	-2	-7	7	59	-11	78	16	-7
**H2 (h)**	-6	-44		-1	-6	2	-3	-21	-82	18	5	-26
**H3 (h)**	-2	12		-4	-8			-1		87	-7	-14
**5-HT1A (h)**	28	92		33	35	-6	0	24	2	74	26	21
**5-HT1B**						-7	-11		-10			
**5-HT1D**			31									
**5-HT2A (h)**	0	95		33	56	-2	16	49	-21	67	70	22
**5-HT2B (h)**	70	96	16	28	94	3	28	29	87	93	65	22
**5-HT2C (h)**						-3	8		10			
**5-HT3 (h)**	33	15		0	12	3	18	-6	-5	13	-11	12
**5-HT4e**			12									
**5-HT5a (h)**						-1	41		43			
**5-HT6 (h)**						-3	1		2			
**5-HT7 (h)**						-6	-13		4			
**5-HT transporter (h)**	-17	23		-5	-18	5	3	22	-11	11	3	-35
**I1**	24	2		3	9			-2		12		5
**IP (PGI2) (h)**						-6	17		32			
**kappa (KOP)**	79	74		94	53	23	-3	99	0	87	57	25
**KV channel**						-13	-7		4			
**LPA1 (Edg-2)**			25									
**M1 (h)**						-13	-32	-4	-29		-5	
**M2 (h)**	6	25		42	-10	-14	-11		2	49	1	-20
**M3 (h)**						6	21		30			
**M4 (h)**	12	55		33	14	-11	-11	49	9	74		22
**M5 (h)**						-3	-5		4			

**MC1**			10									
**MC3**			53									
**MC4 (h)**						-10	1		13			
**MC5**			39									
**MCH1**			-12									
**MCH2**			-3									
**motilin**			25									
**mu (MOP) (h)**	21	57		93	7	7	7	102	23	46	21	11
**MT1 (ML1A) (h)**			62			-4	-5		52			
**MT3 (ML2)**	69	22	4	-2	49			33		49		-21
**Na+ channel (site 2)**	68	71		57	50	-20	30	40	5	87		34
**N neuronal alpha 4beta 2 (h)**											-2	
**N muscle-type (h)**	7	-4		12	-12			-2		38	5	-3
**NK1 (h)**						13	29		42			
**NK2 (h)**						36	68		31			
**NK3 (h)**						-1	13		24			
**NOP (ORL1) (h)**						-5	-5		4			
**norepinephrine transporter (h)**	33	89		56	39	-9	33	59	58	26	38	29
**NTS1 (NT1) (h)**						0	-23		-19			
**P2X**						-5	2		-10			
**P2Y**						3	1		-7			
**PAC1 (PACAP) (h)**						12	-3		0			
**PCP**	-3	1		-1	-19	2	-9	-1	-25	2	9	-13
**PDGF**						-12	-28		10			
**PPARα (h)**	-4		-5	-2	-16	-25	-21	-9	-28	-18	-17	-14
**PPARgamma (h)**	39	85		55	67	-5	14	86	17	23	99	31
**PR**			12									
**sigma (non-**	53	60		59	8	2	9	51	-17	101		36

**selective) (h)**												
**SKCa channel**						-13	-4		16			
**sst (non-selective)**						-20	-6		15			
**sst4 (h)**	64	63		51	35			38		63		25
**TNF-alpha(h)**									8			
**TR (TH)**			-2									
**VDR**			9									
**TP (h) (TXA2/PGH2)**						4	8					
**V1a (h)**						6	6		16			
**VPAC1 (VIP1) (h)**						-32	-24		-65			
**Y1 (h)**						-25	-21		-2			
**Y2 (h)**						-6	-12		9			
Enzyme and cell-based assays												
**Abl kinase (h)**											8	
**ACE (h)**	-31	-21		-21	1			-52		6	-30	46
**acetylcholinesterase (h)**	3	-5		-12	0			-3		3	-7	-7
**CDK2 (h) (cycA)**	-6	-15		-11	-7			-8		-17	-2	-6
**COX2 (h)**	50	20		30	1			11		45	96	-1
**GSK3alpha (h)**	-1	-2		-7	-5			-9		-12	-1	-2
**GSK3beta (h)**	-13	-6		-7	-13			-5		-14	-7	-13
**HIV-1 protease**	27	3		31	4			19		-12	14	22
**MAO-A (h)**	15	0		12	13			-3		6	-12	19
**MAO-B (h) recombinant enzyme**	-4	-18		11	-6			21		18		-9
**MMP-9 (h)**											-32	
**PDE3B (h)**											-2	
**PDE4D2 (h)**											12	
**PDE5 (h) (non-selective)**	-17	-5		-17	19			16		-13		71
**xanthine**	-1	2		-1	9			2		5	6	-1

**oxidase/ superoxide O2- scavenging**												
**ZAP70 kinase (h)**	0	-25		1	9			10		-3	-5	-4

Supplementary Methods

Operational analysis for determination of biased signaling

Step-by-step protocol to calculate bias using the nonlinear regression curve fitting program Graphpad Prism 6.0

Data collection

1) Measure dose-response curves of ligands of interest on assays of interest and normalize to the effect of 10 µM CP55940, or collect previously measured data.
2) Select a reference compound. This compound should be a full agonist in all assays under consideration with similar potency.

Data analysis

1) Put the data for all ligands that act as full agonists in column A-O. If you have more than 15 full agonists, use a second table, or adjust the equation depicted below. If you have fewer than 15 full agonists, leave the remaining columns blank.
2) Insert the data of partial agonists from column P onwards.
3) Create a user-defined equation:

   Choose – Analyze→Non-linear regression (curve fit)→New→Create New Eqn

Equation tab:	Equation Type -	Explicit Equation: Y = a function of X and parameters
	Name -	Operational Model for Bias

Definition - A=10^X

operate1=((1+A)/((10^logR)*A))^n

operate2=((1+A/(10^logKA))/((10^LogR)*A))^n

Y1=basal+(Emax-basal)/(1+operate1)

Y2=basal+(Emax-basal)/(1+operate2)

<A:O>Y=Y1

<~A:O>Y=Y2

Rules for initial values tab:

LogR	-1.0	*(Value of X at YMID)
n	1.0	
LogKA	1.0	*(Value of X at YMID)
basal	1.0	*YMIN
Emax	1.0	*YMAX

Default range: start graphing the curve at the smallest X value

Default constraints tab:

LogR	no constraint
n	Shared value for all datasets
LogKA	no constraint

Basal	Shared value for all datasets
Emax	Constant equal to 100

4) Analyze all dose-response data using the Operational model equation

Analyze→Non-liinear regression (curve fit)→User-defined equation→ Operational Model for Bias

If the curve fit is ambiguous, change the constraints and initial values, e.g. setting the slope “n=1”, the basal to “0” (only if the baseline is substracted), or the Emax to “no constraint” or “shared for all datasets”

5) Take the logR (equivalent to Log (τ/KA)) and the SE LogR values from the results sheet.

Calculation of ΔlogR

1) Calculate ΔlogR values for the ligands per assay using a reference ligand (ref) using equation 1, according to the example shown below. Pth=pathway

$$\Delta LogR_{ligand:pth} = LogR_{ligand:pth} - LogR_{ref:pth} \qquad \text{Eq. 1}$$

The example shown has CP55940 as the reference compound, WIN55212-2 as the ligand of interest and pERK as the used assay. LogR values can be found in Table S2.

$$\Delta LogR_{WIN55212-2:pERK} = LogR_{WIN55212-2:pERK} - LogR_{CP55940:pERK}$$

$$\Delta LogR_{WIN55212-2:pERK} = 9.29 - 9.03$$

$$\Delta LogR_{WIN55212-2:pERK} = 0.26$$

2) Calculate the SEM for the ΔlogR using equation 2, according to the example shown below.

$$SE\Delta LogR_{ligand:pth} = \sqrt{(SELogR_{ligand:pth})^2 + (SELogR_{ref:pth})^2} \qquad \text{Eq. 2}$$

The example shown has CP55940 as the reference compound, WIN55212-2 as the ligand of interest, and pERK as the used assay. SELogR values are shown in Table S2.

$$SE\Delta LogR_{WIN55212-2:pERK} = \sqrt{(SELogR_{WIN55212-2:pERK})^2 + (SELogR_{CP55940:pERK})^2}$$

$$SE\Delta LogR_{WIN55212-2:pERK} = \sqrt{(0.17)^2 + (0.17)^2}$$

$$SE\Delta LogR_{WIN55212-2:pERK} = \sqrt{0.0289 + 0.0289}$$

$$SE\Delta LogR_{WIN55212-2:pERK} = \sqrt{0.0578}$$

$$SE\Delta LogR_{WIN55212-2:pERK} = 0.24$$

ΔlogR of WIN55212-2 on pERK: 0.26 ± 0.24

3) Calculate relative effectiveness (RE) of each ligand using equation 3

$$RE_{ligand:pth} = 10^{\Delta LogR} \qquad \text{Eq. 3}$$

Example: Relative effectiveness of WIN55212-2 on pERK

$RE_{WIN55212-2:pERK} = 10^{0.26}$

$RE_{WIN55212-2:pERK} = 1.8$

Calculation of ΔΔlogR and bias factors

1) Calculate the ΔΔlogR for all ligands to to determine differences in relative effectiveness between pathways. ΔΔlogR values can be calculated using equation 4, according to the example shown below.

$$\Delta\Delta LogR_{ligand:pth1-pth2} = \Delta LogR_{ligand:pth1} - \Delta LogR_{ligand:pth2} \qquad \text{Eq. 4}$$

In the example shown is WIN55212-2 the ligand of interest, and pERK and GIRK as the pathways that are compared. ΔlogR values can be found in table S2.

$\Delta\Delta LogR_{WIN55212-2:pERK-GIRK} = \Delta LogR_{WIN55212-2:pERK} - \Delta LogR_{WIN55212-2:GIRK}$

$\Delta\Delta LogR_{WIN55212-2:pERK-GIRK} = 0.26 - -0.25$

$\Delta\Delta LogR_{WIN55212-2:pERK-GIRK} = 0.51$

2) Calculate the SEM for the ΔΔlogR values using equation 5, according to the example shown below.

$$SE\Delta\Delta LogR_{ligand:pth1-pth2} = \sqrt{(SE\Delta LogR_{ligand:pth1})^2 + (SE\Delta LogR_{ligand:pth2})^2} \quad \text{Eq. 5}$$

In the example shown is WIN55212-2 compared between pERK an GIRK. The SEΔlogR values can be found in Table S2.

$$SE\Delta\Delta LogR_{WIN:pERK-GIRK} = \sqrt{(SE\Delta LogR_{WIN:pERK})^2 + (SE\Delta LogR_{WIN:GIRK})^2}$$

$$SE\Delta\Delta LogR_{WIN:pERK-GIRK} = \sqrt{(0.24)^2 + (0.10)^2}$$

$$SE\Delta\Delta LogR_{WIN:pERK-GIRK} = \sqrt{0.0576 + 0.01}$$

$$SE\Delta\Delta LogR_{WIN:pERK-GIRK} = 0.26$$

The ΔΔlogR of WIN55212-2 between pERK and GIRK: 0.51 ± 0.26

3) The bias factor for a ligand between pathways is the inverse log of the ΔΔlogR of a given ligand between two given pathways (equation 6).

$$BF_{ligand:pth1-pth2} = 10^{\Delta\Delta LogR} \quad \text{Eq. 6}$$

Thus, for WIN55212-2 between pERK and GIRK:

$$BF_{WIN:pERK-GIRK} = 10^{0.51}$$

$$BF_{WIN:pERK-GIRK} = 3.24$$

Synthesis of Δ^{9}-THC

BF_3Et_2O catalyzed electrophilic aromatic substitution of Olivetol with chiral monoterpene **1**, in dry conditions, yielded Δ^{9}-THC in one step (see scheme **1**).[40,41]

Scheme 1. Synthesis of Δ^{9}-THC

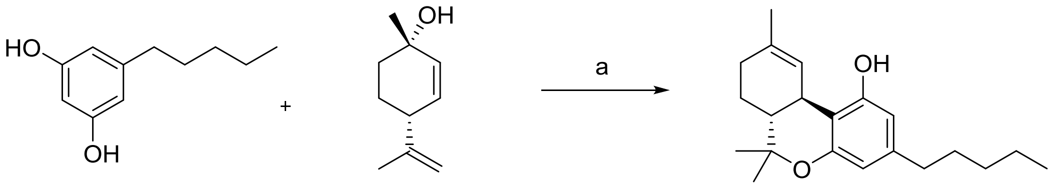

Olivetol 1 **2,** Δ^{9}-THC

Reagents and conditions: a) BF_3Et_2O, $MgSO_4$, DCM, 0°C to rt

Synthetic procedure to (-)-Δ^{9}-tetrahydrocannabinol 2

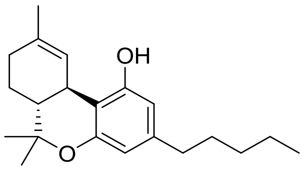

A flame-dried 10 ml round bottom flask was charged with a magnetic stirring bar, olivetol (180 mg, 1 mmol, 1 eq), and purged with Ar. Dry DCM (1 ml) was added, along with anhydrous $MgSO_4$ (375 mg, 3.1 mmol, 3.1 eq), and the flask was purged with Ar again. Monoterpene **1** (167 mg, 1.1 mmol, 1.1 eq), in dry DCM (2 ml), was added, and the flask was cooled to 0°C in an ice water bath. BF_3Et_2O (65 µl, 0.5 mmol, 0.5 eq) was added dropwise, and the reaction was stirred at 0°C for 3 h. Upon completion the reaction was quenched with anhydrous $NaHCO_3$ (1 g). The reaction was allowed to stir for an additional 30 min, resulting in progressive loss of color, upon which the reaction was filtered through a pad of

celite, and the filtrate concentrated. After concentration, the crude residue (~350 mg) was first purified by flash column chromatography (1 to 4% Et_2O in pentane), then purified by preparative HPLC chromatography, yielding the product as a light yellow oil (35 mg, 0.11 mmol, 11%) as a clear, viscous oil. Rf: 0.8 (10% Et_2O/pentane). ^{1}H NMR (400 MHz, $CDCl_3$) δ 6.30 (s, 1H), 6.27 (d, J = 1.1 Hz, 1H), 6.14 (d, J = 1.3 Hz, 1H), 4.97 – 4.31 (m, 1H), 3.20 (d, J = 10.9 Hz, 1H), 2.43 (dd, J = 8.4, 6.3 Hz, 2H), 2.20 – 2.11 (m, 2H), 1.91-1.69 (m, 2H), 1.68 (s, 3H), 1.58 – 1.52 (m, 2H), 1.41 (s, 3H), 1.36 – 1.24 (m, 4H), 1.09 (s, 3H), 0.88 (5, J = 7.6, 3H) ppm. ^{13}C NMR (100 MHz, CDCl3) δ 154.8, 154.2, 142.8, 134.4, 123.9, 110.1, 109.0, 107.5, 77.2, 45.8, 35.5, 33.6, 31.5, 31.2, 30.7, 27.6, 25.0, 23.4, 22.6, 21.7, 19.3, 14.0 ppm. HRMS (ESI+) m/z: calculated for $C_{21}H_{30}O_2$ [M + H]+: 315.2279, found 315.2319.

Synthesis of JWH015

2-Methylindole **1** was N-alkylated with 1-bromopropane using NaH to yield 2-methyl-1-indole **2** in quantitative yield. 1-Napthoic acid was converted into its corresponding acid chloride **4** using thionyl chloride. Friedel-Crafts acylation of 2-methyl-1-indole **2** with 1-naphtoyl chloride **4** using diethylammonium chloride yielded JWH015 **5** (see scheme **2**).[42]

Scheme 2. Synthesis of JWH015

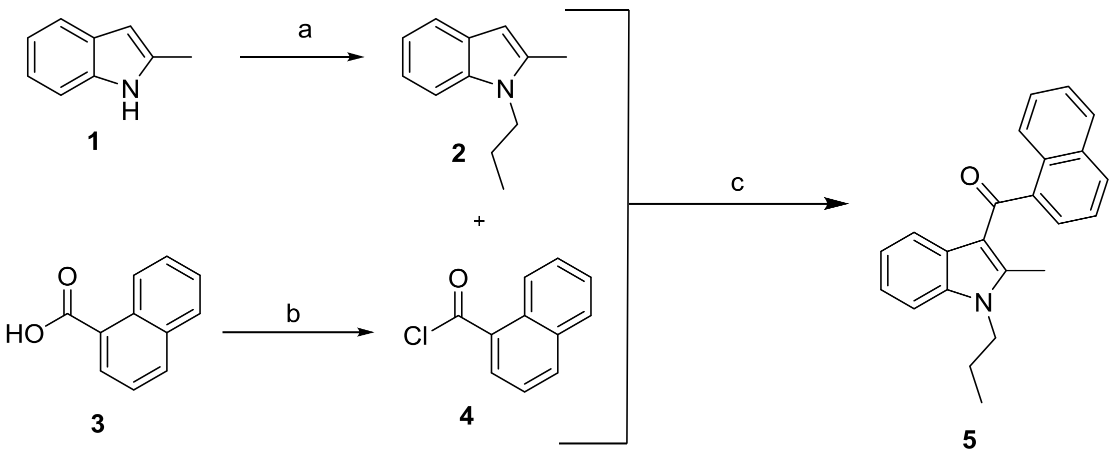

Reagents and conditions: a) NaH, 1-bromopropane, DMF, 0°C to rt; b) thionyl chloride, THF, 50°C; c) diethylaluminium chloride, DCM, 0°C

Synthetic procedure to 2-methyl-1-propylindole 2

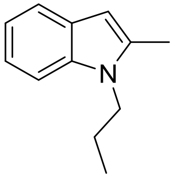

2-Methylindole (0.262 g, 2 mmol, 1eq) was dissolved in DMF (10 ml) and cooled to 0 °C. NaH (0.120 g, 3.00 mmol, 1.5 eq) was added portionwise, and after that 1-bromopropane (0.218 ml, 2.400 mmol, 1.2 eq) was added. After stirring for 13.50 hr at 0°C, MeOH was added to quench the remaining NaH. The mixture was concentrated in vacuo. EtOAc and brine were added to the remaining solution and the layers were separated. The waterlayer was extracted with EtOAc (3x) and the organic layers were washed with brine, dried with MgSO4, filtered, and concentrated in vacuo, yielding an orange oil (0.338 g, 1.951 mmol, 98%). The product was continued without further purification. ^{1}H NMR (400 MHz, Chloroform-*d*) δ 7.51 (d, *J* = 7.7 Hz,

1H), 7.29 – 7.23 (m, 1H), 7.17 – 7.09 (m, 1H), 7.05 (t, *J* = 7.4 Hz, 1H), 6.22 (s, 1H), 4.02 (t, *J* = 7.4 Hz, 2H), 2.55 – 2.36 (m, 3H), 1.78 (h, *J* = 7.4 Hz, 2H), 0.95 (t, *J* = 7.4 Hz, 3H).

Synthetic procedure to 1-naphthoyl chloride 4

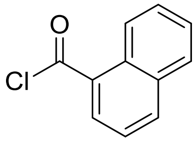

1-naphthoic acid (0.359 g, 2 mmol, 1 eq) was dissolved in anhydrous THF (10 ml). Thionyl chloride (0.219 ml, 3.00 mmol, 1.5 eq) was added and the mixture was stirred at 50 °C for 19.00 hr. The mixture was concentrated in vacuo under an argon atmosphere and the dark green oil was used in the next step without further purification.

Synthesis of JWH015

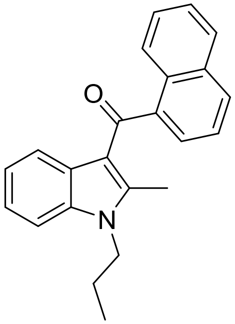

2-methyl-1-propyl-1H-indole **2** (0.347 g, 2 mmol, 1 eq) was dissolved in DCM (2 ml) and diethylaluminum chloride (DAC, 1.667 ml, 3.00 mmol, 1.5 eq) was added. The mixture was cooled to 0 °C and 1-naphthoyl chloride **4** (0.381 g, 2.000 mmol, 1 eq), dissolved in 8 mL DCM, was added dropwise. Upon addition of DAC, the mixture turned dark brown. The mixture was allowed to reach rt, and was stirred for 4.50 hr. The reaction was carefully quenched with water and 2 M NaOH was added. The product was extracted with DCM (3x) and the organic layers were washed with brine, dried with MgSO4, filtered and concentrated in vacuo. The product was purified using column chromatography (10 to 15% EtOAc in PE), then purified by preparative TLC (30% EtOAc in PE, Rf: 0.5), yielding the product as a light yellow oil (17 mg, 0.05 mmol, 3%). ^{1}H NMR (400 MHz, $CDCl_3$) δ 8.15 – 8.08 (m, 1H), 7.96 (dt, *J* = 8.1, 1.1 Hz, 1H), 7.90 (dd, *J* = 8.6, 1.3 Hz, 1H), 7.56 (dd, *J* = 7.1, 1.4 Hz, 1H), 7.49 (ddd, *J* = 8.3, 6.8, 1.5 Hz, 2H), 7.42 (ddd, *J* = 8.3, 6.8,

1.4 Hz, 1H), 7.32 – 7.28 (m, 1H), 7.19 – 7.13 (m, 2H), 7.03 – 6.95 (m, 1H), 4.08 (t, *J* = 8.0 Hz, 2H), 2.47 (s, 3H), 1.82 (h, *J* = 7.5 Hz, 2H), 0.98 (t, *J* = 7.4 Hz, 3H). ^{13}C NMR (101 MHz, $CDCl_3$) δ 193.54, 145.73, 140.64, 136.27, 133.92, 130.49, 130.04, 128.32, 127.22, 126.95, 126.34, 125.80, 125.75, 125.20, 122.32, 122.02, 121.35, 115.04, 109.60, 44.97, 23.05, 12.69, 11.56. HRMS (ESI+) m/z: calculated for $C_{23}H_{22}NO$ $[M + H]^+$: 328.16959, found 328.16939.

Supplementary References

1. Baggelaar, M. P. *et al.* Development of an activity-based probe and in silico design reveal highly selective inhibitors for diacylglycerol lipase-α in brain. *Angew. Chemie - Int. Ed.* **52,** 12081–12085 (2013).
